# Supplementary material for: MR Spectroscopy Without Water Suppression Using the Gradient Impulse Response Function
Source: Magn Reson Med. 2026 May 5;96(2):530–41. doi: 10.1002/mrm.70383 (PMC13269187; doi:10.1002/mrm.70383)
Supplement: Supplementary file 1 — Table S1: Voxel locations, orientations and rotations for all participant and phantom acquisitions. Voxel prescription parameters are reported in the Siemens patient‐based coordinate system for a head‐first supine patient position. A, anterior; C, coronal; F, foot; H, head; L, left; P, posterior; R, right; T, transverse. Figure S1: Unfitted spectra for all participants following water removal for (A) Semi‐LASER acquisitions and (B) MEGA‐PRESS acquisitions. Figure S2: Fitted spectra for all participants for (A) Semi‐LASER acquisitions and (B) MEGA‐PRESS acquisitions. The fitted spectral baselines are also shown and have been vertically offset for visual clarity. Figure S3: Fitted spectra for all participants before and after system heating in the semi‐LASER acquisitions. The fitted spectral baselines are also shown and have been vertically offset for visual clarity. [file MRM-96-530-s001.pdf]

Supporting Information

	Semi-LASER		MEGA-PRESS	
**Participant**	**Voxel Position (mm)**	**Voxel Orientation and Rotation**	**Voxel Position (mm)**	**Voxel Orientation and Rotation**
**001**	L: 34.7<br>P: 8.7<br>H: 47.2	Orientation: Coronal<br>Rotation: 0°	L: 1.0<br>P: 56.5<br>F: 3.0	Orientation: T>C 36.9°<br>Rotation: 0°
**002**	L: 39.8<br>A: 9.1<br>H: 24.2	Orientation: Coronal<br>Rotation: 0°	L: 3.7<br>P: 38.5<br>F: 17.6	Orientation: T>C 38.7°<br>Rotation: -180°
**003**	L: 32.7<br>P: 2.1<br>H: 54.8	Orientation: Coronal<br>Rotation: 0°	L: 0.0<br>P: 42.2<br>H: 14.8	Orientation: C>T 43.4°<br>Rotation: 90°
**004**	L: 29.7<br>P: 13.9<br>H: 34.6	Orientation: Coronal<br>Rotation: 0°	R: 3.1<br>P: 55.9<br>H: 0.1	Orientation: T>C36.9°<br>Rotation: 0°
**005**	L: 29.6<br>P: 16.7<br>H: 50.2	Orientation: Coronal<br>Rotation: 0°	L: 2.3<br>P: 59.1<br>H: 4.6	Orientation: T>C 36.9°<br>Rotation: 0°
**006**	L: 31.9<br>A: 6.7<br>H: 56.8	Orientation: Coronal<br>Rotation: 0°	L: 2.4<br>P: 35.3<br>H: 18.6	Orientation: C>T 36.1°<br>Rotation: 0°
**007**	L: 28.3<br>P: 19.6<br>H: 30.4	Orientation: Coronal<br>Rotation: 0°	R: 1.2<br>P: 54.6<br>F: 10.9	Orientation: T>C 34.7°<br>Rotation: 0°
**008**	L: 33.2<br>A: 3.2<br>H: 46.7	Orientation: Coronal<br>Rotation: 0°	L: 2.0<br>P: 36.6<br>H: 14.5	Orientation: C>T 39.5°<br>Rotation: 90°
**Phantom**	L: 29.7<br>P: 13.9<br>H: 34.6	Orientation: Coronal<br>Rotation: 0°	R: 3.1<br>P: 40.9<br>H: 0.1	Orientation: T>C 36.9°<br>Rotation: 0°

Supporting Information Table S1: Voxel locations, orientations and rotations for all participant and phantom acquisitions. Voxel prescription parameters are reported in the Siemens patient-based coordinate system for a head-first supine patient position. Abbreviations: L, left; R, right; A, anterior; P, posterior; H, head; F, foot; C, coronal; T, transverse.

A)

Semi-LASER: Unfitted Metabolite Spectra

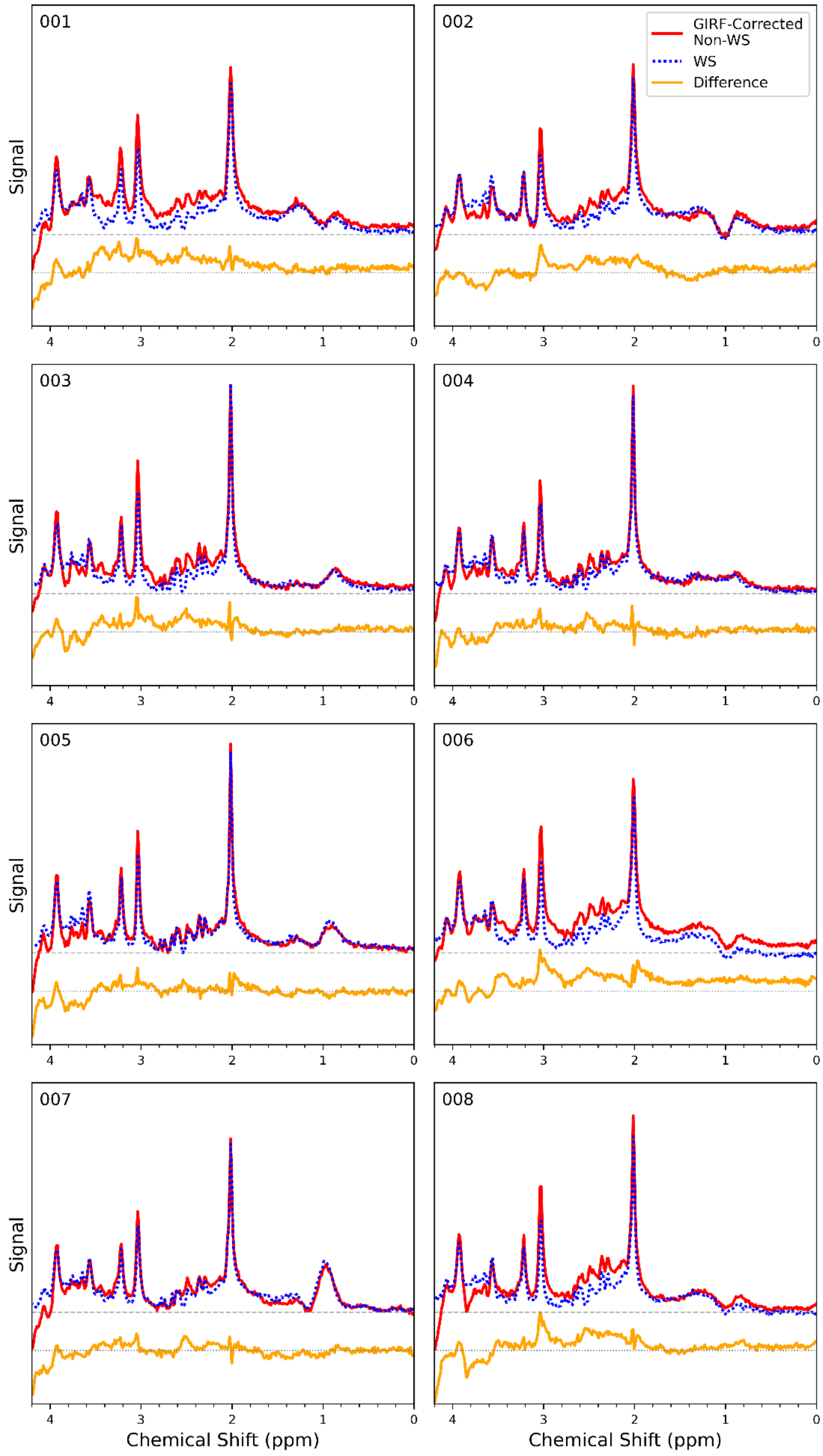

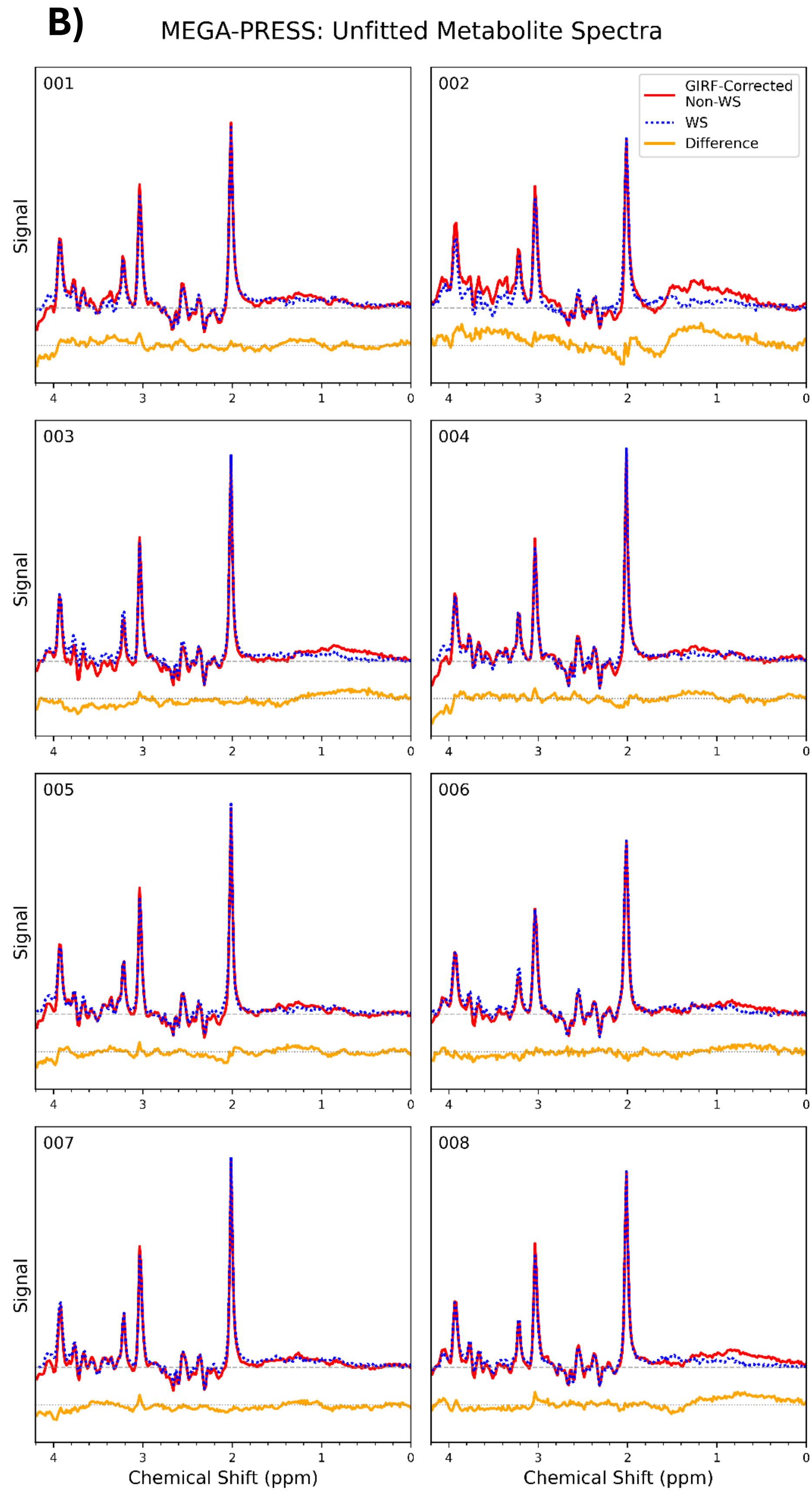

Supporting Information Figure S1: Unfitted spectra for all participants following water removal for A) Semi-LASER acquisitions and B) MEGA-PRESS acquisitions.

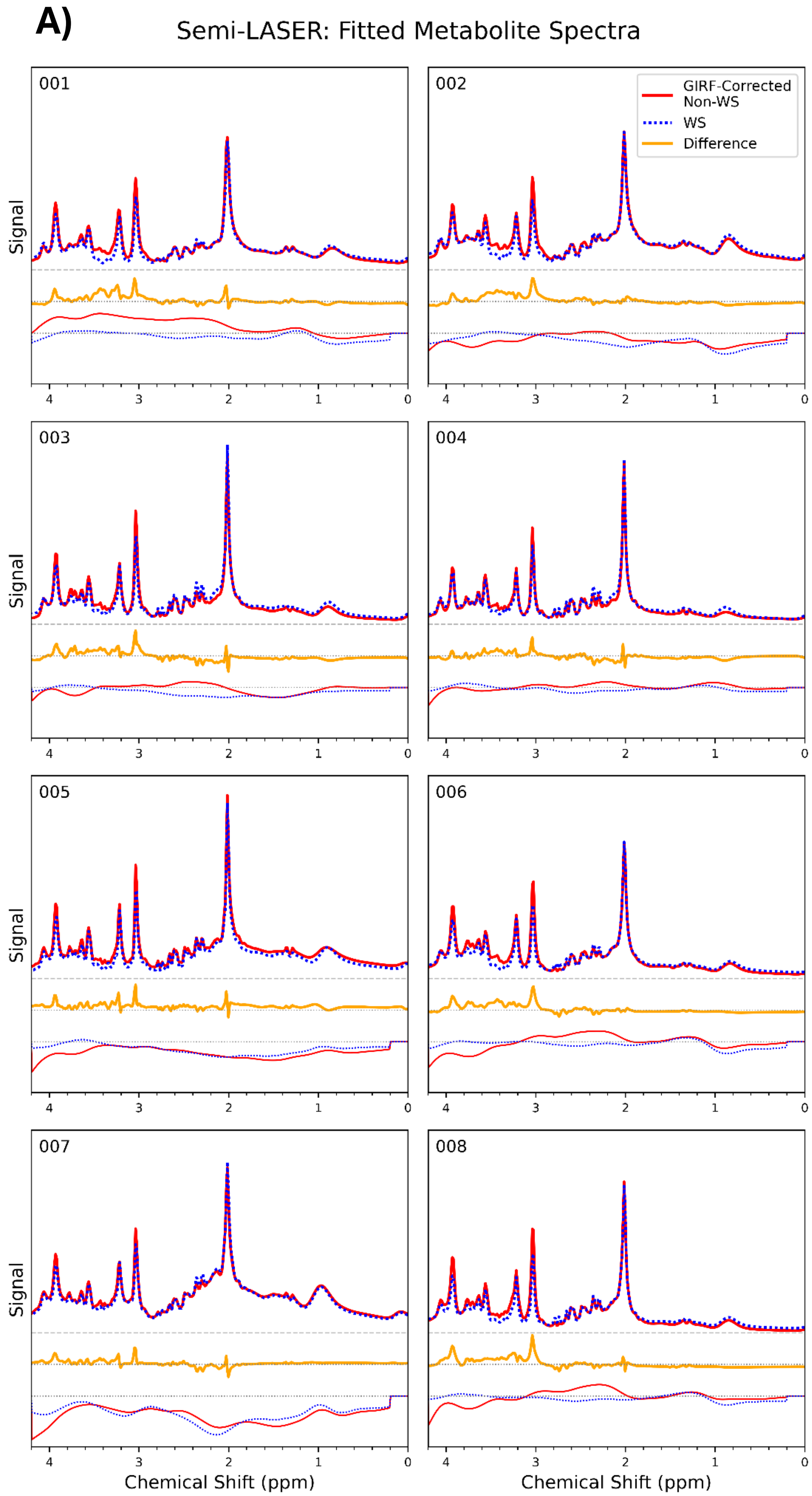
A)
Semi-LASER: Fitted Metabolite Spectra
001
002
003
004
005
006
007
008
GIRF-Corrected Non-WS
WS
Difference
Signal
Chemical Shift (ppm)

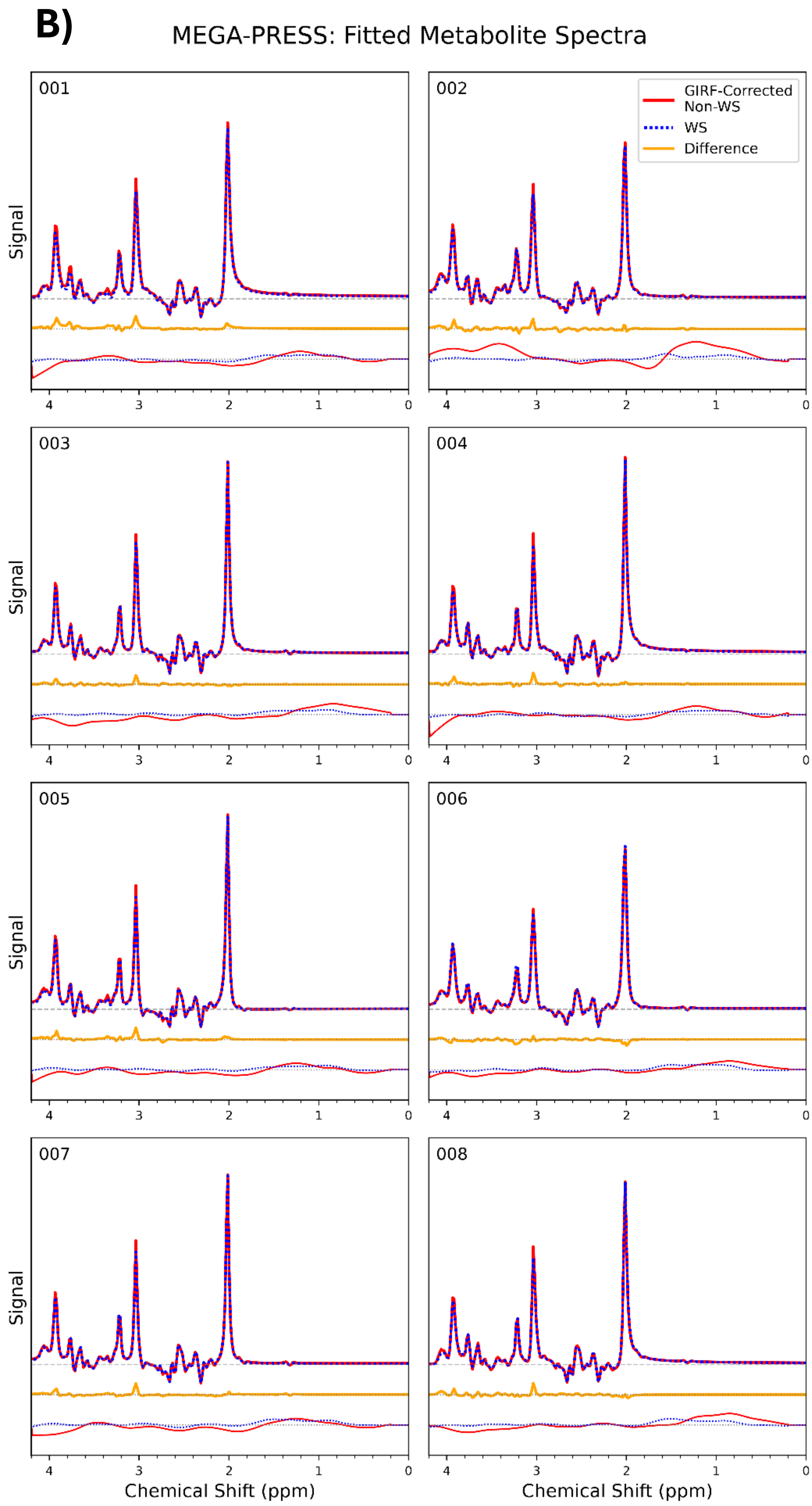

Supporting Information Figure S2: Fitted spectra for all participants for A) Semi-LASER acquisitions and B) MEGA-PRESS acquisitions. The fitted spectral baselines are also shown and have been vertically offset for visual clarity.

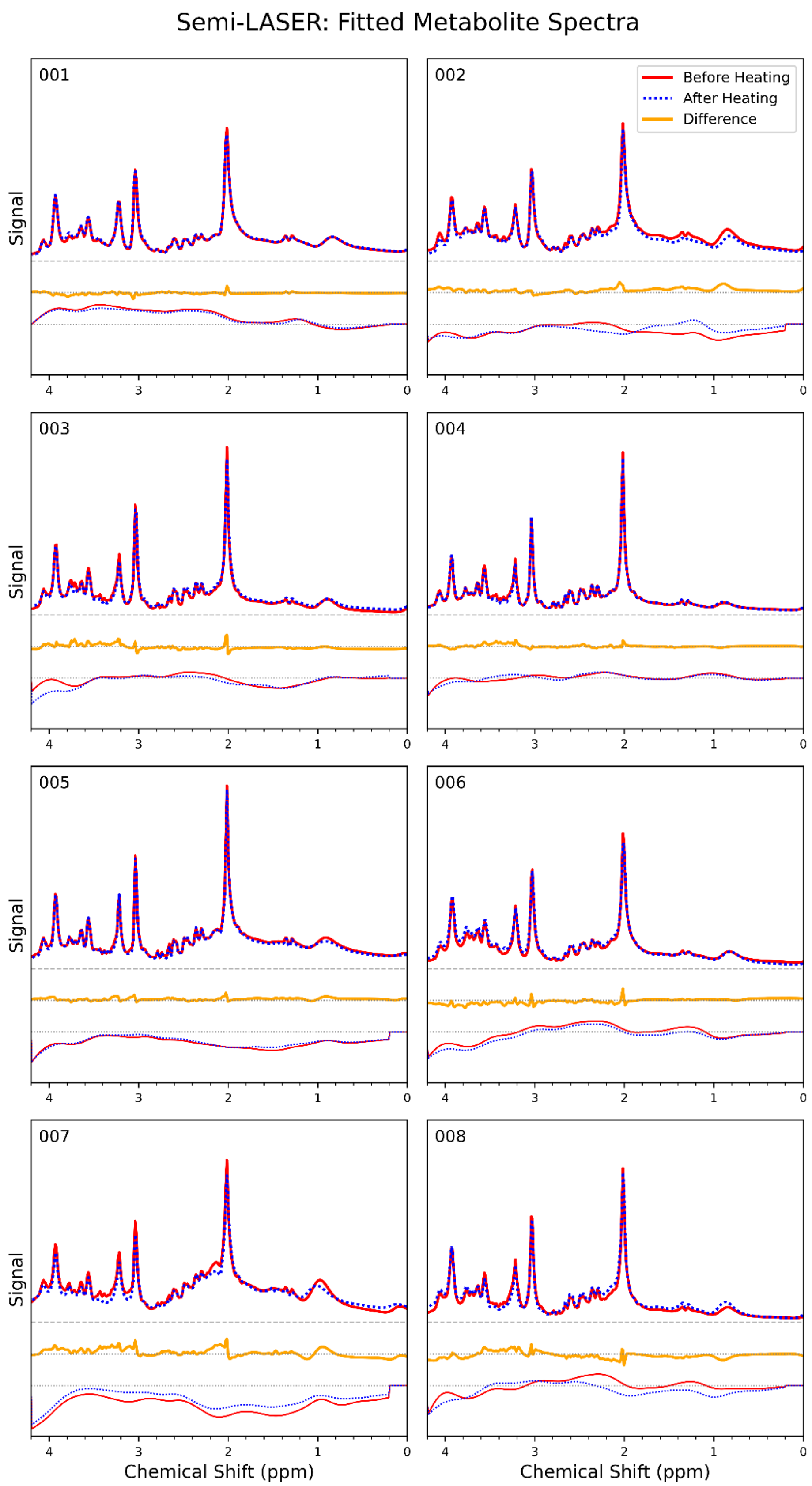

Supporting Information Figure S3: Fitted spectra for all participants before and after system heating in the semi-LASER acquisitions. The fitted spectral baselines are also shown and have been vertically offset for visual clarity.
